# Retraction: Messina et al. Rationale and Criteria for a COVID-19 Model Framework. *Viruses* 2021, *13*, 1309

**DOI:** 10.3390/v13081485

**Published:** 2021-07-29

**Authors:** Viruses Editorial Office

**Affiliations:** MDPI, St. Alban-Anlage 66, 4052 Basel, Switzerland; viruses@mdpi.com

The journal retracts the 6 July 2021 article [1].

Following publication, concerns were brought to the attention of the publisher regarding improper reuse of text.

Adhering to our complaints procedure, an investigation was conducted in which we confirmed the extent of the overlap, which included Figure 1, Table 4, the conclusions, and supplementary material, and the article is therefore retracted.

This retraction was approved by the Editor in Chief of the journal *Viruses*.

The authors agreed to this retraction.
